# The Preoperative Diagnostic Performance of Multi-Parametric Quantitative Assessment in Rectal Carcinoma: A Preliminary Study Using Synthetic Magnetic Resonance Imaging

**DOI:** 10.3389/fonc.2022.682003

**Published:** 2022-05-25

**Authors:** Kexin Zhu, Zhicheng Chen, Lingling Cui, Jinli Zhao, Yi Liu, Jibin Cao

**Affiliations:** ^1^ Department of Radiology, The First Affiliated Hospital of China Medical University, Shenyang, China; ^2^ Department of Radiology, Shengjing Hospital of China Medical University, Shenyang, China

**Keywords:** rectal carcinoma, extramural vascular invasion, EMVI, Synthetic Magnetic Resonance Imaging, Synthetic MRI

## Abstract

**Objective:**

Synthetic MRI (SyMRI) can reconstruct different contrast-weighted images(T1, T2, PD) and has shorter scan time, easier post-processing and better reproducibility. Some studies have shown splendid correlation with conventional mapping techniques and no degradation in the quality of syMRI images compared with conventional MRI. It is crucial to select an individualized treatment plan based on the preoperative images of rectal carcinoma (RC). We tried to explore the feasibility of syMRI on T, N stage and extramural vascular invasion (EMVI) of rectal cancer.

**Materials and Methods:**

A total of 100 patients (37 females and 63 males) diagnosed with rectal carcinoma were enrolled. All the patients underwent preoperative pelvic MR examinations including conventional MR sequence and synthetic MRI. Two radiologists evaluated the MRI findings of each rectal carcinoma and EMVI score in consensus. The values for T1, T2 relaxation times and PD value were measured in tumor(ROI-1) and pararectal fat space(ROI-2) and analyzed independently. A receiver operating characteristic (ROC) analysis was performed. Correlations between the T1, T2 and PD values and EMVI score were also evaluated.

**Results:**

Compared with the normal rectal wall, the values of T1 and T2 relaxation times of the tumor were significantly higher (*P <*0.001). There was no statistically significant difference in the PD value (P >0.05). As for ROI, the ROI of pararectal fat space(ROI-2) had better significance than rectal cancer lesion (ROI-1). T2 value of ROI-1 and T1 value of ROI-2 were higher in the pEMVI positive group than in the negative group (P=0.002 and 0.001) and T1 value of ROI-2 had better performance with an AUC of 0.787, (95% CI:0.693- 0.882). T1 value, T2 value and PD value from ROI-2 were effective for both T and N stage of rectal cancer. High-grade pathological stage had showed higher T1 value (P_T stage_=0.013,P_N stage_=0.035), lower T2 value (P_T stage_=0.025,P_N stage_=0.034) and lower PD value (P_T stage_=0.017). We also enrolled the characteristics with P < 0.05 in the combined model which had better diagnostic efficacy. A significant positive correlation was found between the T1 value of pararectal fat space(ROI-2) and EMVI score (r value = 0.519, P<0.001). The T2 value(r=0.213,P=0.049) and PD value(r=0.354,P=0.001) from ROI-1 was correlated with EMVI score. Correlation analysis did not show any significant associations between T2 value of tumor, T2, PD values of pararectal fat space and EMVI scores.

**Conclusion:**

Synthetic MRI can provide multi-parameter quantitative image maps with a easier measurement and slightly shorter acquisition time compared with conventional MRI. The measurement of multi-parametric quantitative values contributes to diagnosing the tumor and evaluating T stage, N stage and EMVI. It has the potential to be used as a preoperative diagnostic and grading technique in rectal carcinoma.

## Introduction

Rectal carcinoma (RC) is the fourth most common cancer and also the second leading cause of cancer death worldwide (1). The incidence of rectal carcinoma is continually increasing in China (2). The preoperative image diagnosis of rectal carcinoma is crucial for tailoring an individualized treatment plan (3).The preoperative MRI imaging evaluation of rectal carcinoma included lesion location (the distance from the anal verge), T stage, N stage, extramural vascular invasion (EMVI), and mesorectal fascia (MRF) involvement (4). Anal examination combined with conventional high-resolution pelvic MRI examination is routinely used to evaluate the rectal carcinoma invasiveness (5).

However, the morphologic changes in rectal carcinoma accessed by conventional MRI are susceptible to the observer bias (6) and image quality (7).In recent years, multiparametric MRI including diffusion-weighted imaging (DWI) (8, 9), intravoxel incoherent motion (IVIM) (10), diffusion kurtosis imaging (DKI) (11), and dynamic contrast-enhanced (DCE) (12),and radiomics model of MRI have been applied to predict the pathological factors of RC. However, DWI’s susceptibility to artifacts (13), as well as excessively long scan times, cumbersome post-processing procedures, and poor reproducibility limit the clinical use of these techniques (14).

In the past few years, quantitative MRI (qMRI) has been developed rapidly what can offer standardized measurement of specific physical parameters of tissue microstructure, and qMRI indicators are sensitive to multiple biological factors (15). In the background, a multi-parameter mapping (MPM) protocol using multiple parameters simultaneously has emerged. The qMRI sequence based on MPM, what is named as quantification of relaxation times and proton density by multiecho acquisition of a saturation-recovery using turbo spin-echo readout (QRAPMASTER) (16), can yield data such as longitudinal relaxation rate (R1), effective transverse relaxation rate (R2*), and effective proton density (PD*) (8). This technique can reconstruct contrast-weighted images (relaxation maps), which is known as synthetic MRI (17, 18), is able to obtain quantitative parameters (T1, T2, PD, R1, R2) and shorter scan time, easier post-processing, better reproducibility.

In recent years, longitudinal relaxation times (T1 mapping) are often used for the assessment of myocardial injury (6, 19, 20), and transverse relaxation times (T2 mapping) for the quantitatively analyze intervertebral disc and cartilage lesions (21). Previously, syMRI has been successfully applied to studies of the skull (18), prostate (14) and cervical cancer (22), and some studies has shown splendid correlation with conventional mapping techniques and no degradation in the quality of syMRI images compared with conventional MRI. The study by Zhao et al. (23) also showed that syMRI can be used for rectal cancer studies. So, in this study, we tried to explore the feasibility of SyMRI on T, N stage and extramural vascular invasion (EMVI) of rectal cancer.

## Materials and Methods

### Participants

**Figure 1:**
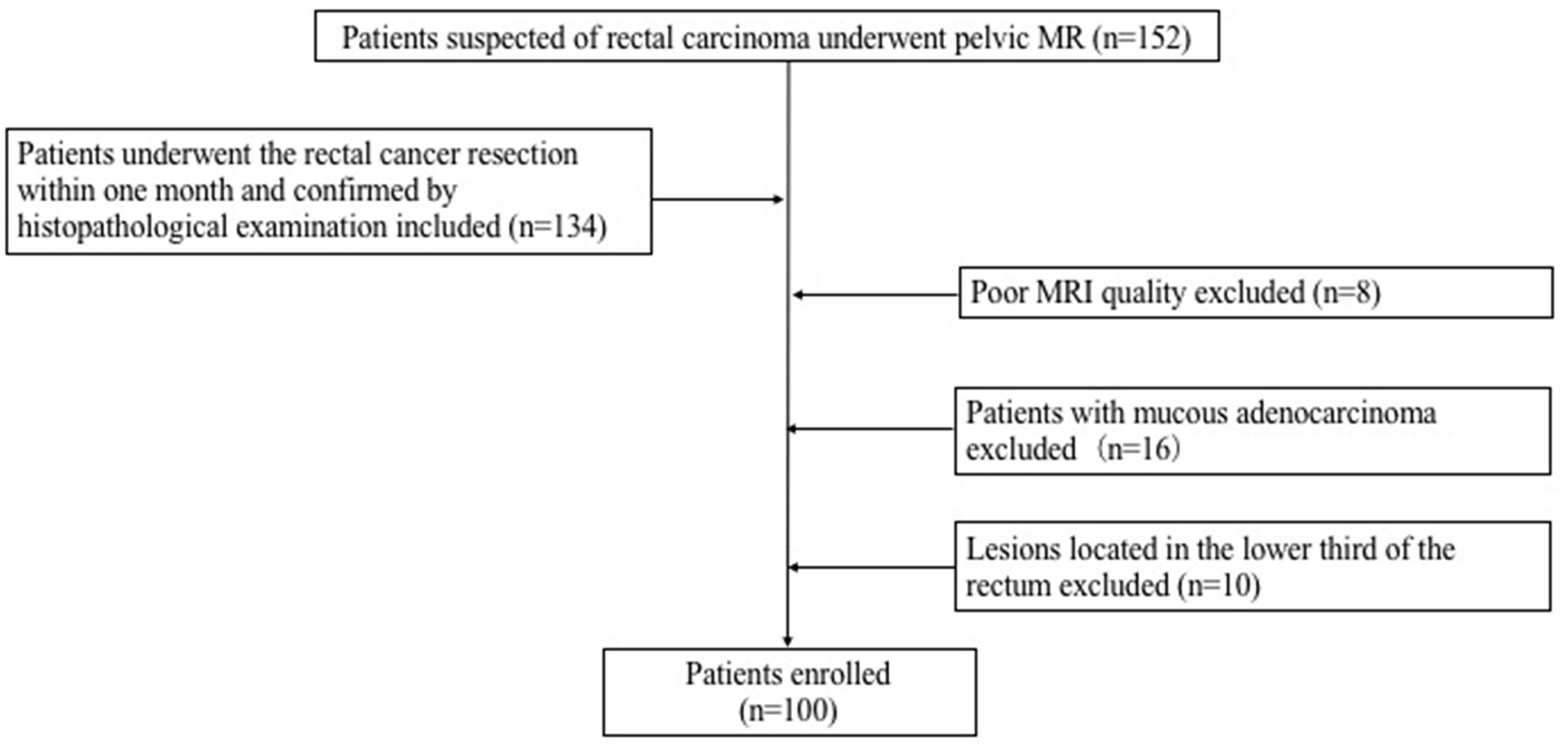
Flowchart for selecting patients.

152 consecutive patients suspected of rectal carcinoma underwent pelvic MR in the First Hospital of China Medical University from December 2018 to December 2020. A total of 100 patients (37 females and 63 males) diagnosed with rectal carcinoma were ultimately enrolled in this study. The inclusion criteria for the patients were as follows: (1) Patients with rectal adenocarcinoma confirmed by histopathological examination; (2) Had no therapies before the MRI examination; (3) All patients underwent the rectal cancer resection within one month after MR examination. Exclusion criteria: (1) The lesions located in the lower third of the rectum; (2) The pathological results were mucinous adenocarcinoma; (3) Poor image quality. The flowchart of this study was shown in **Figure 1**. 32 healthy control (HC) subjects had no history of rectal lesions based on the conventional MRI. This prospective study was approved by our Institutional Review Board. Formal informed consent was ignored and patients verbally committed to cognitive experiments.

### MRI Data

#### MRI Acquisition

All MRI studies were performed on a GE 3.0 T MRI scanner (signa pioneer, General Electric, USA). All the patients underwent pelvic MR examinations including conventional MR sequences and synthetic MR sequence (MAGIC sequence) in a supine position. A multiple-dynamic multiple-echo (MDME) sequence was performed for synthetic MRI: repetition time (TR) = 400ms, echo time (TE)= 17.1ms, 94.3ms, inversion time (TI) = 130, 500, 1370, 2970ms, flip angle=120°, field of view (FOV) = 24 × 24cm, matrix = 320×256, Echo Train Length (ETL) = 16, phase acceleration factor = 2, slice thickness = 4mm, layer spacing = 1mm, 20 slices.

#### MR Image Evaluation and Data Processing

**Figure 2:**
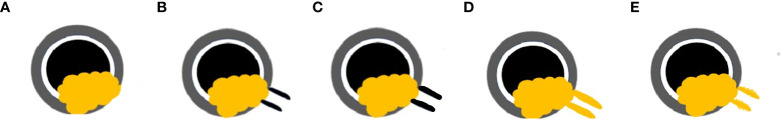
The MRI–detected EMVI (mrEMVI) scoring system. Score 0 **(A)**: Definitely negative(No vessels adjacent to areas of tumor penetration); Score 1 **(B)**: Probably negative (Minimal extramural stranding/nodular extension, but not in the vicinity of any vascular structure); Score 2 **(C)**: Possibly negative (Stranding demonstrated in the vicinity of extramural vessels, but these are of normal caliber, and no definite tumor signal in vessel); Score 3 **(D)**: Probably positive (Intermediate signal intensity apparent within the vessels, although contour and caliber of these vessels are only slightly expanded; Score 4 **(E)**: Definitely positive (Obvious irregular vessel contour or nodular expansion of vessel by definite tumor signal).

Two radiologists with 5 years and 10 years of experience in abdominal MR imaging reviewed all sequences of images in all selected patients with rectal lesions. The MRI findings of each tumor and EMVI scoring were evaluated by two radiologists in consensus. The MRI-detected EMVI (mrEMVI) status was categorized as EMVI-positive and EMVI-negative according to the pathological extramural venous invasion (pEMVI), then the mrEMVI were scored based on the 5-point scale suggested by Smith et al. (24) (**Figures 2A–E**).

**Figure 3:**
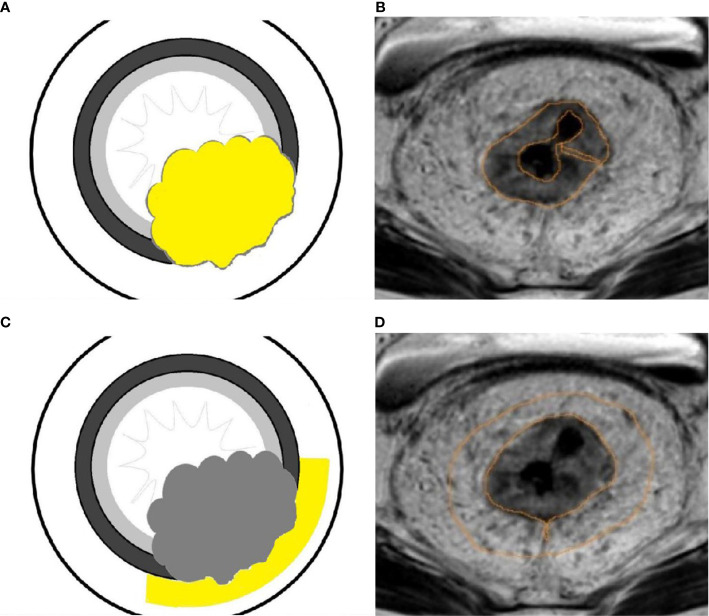
A schematic diagram of ROI delineation for rectal carcinoma **(A)**; Target delineation in rectal carcinoma on the SyMRI image **(B)**; A schematic diagram of ROI delineation for pararectal fat space **(C)**; Target delineation in the pararectal fat space on SyMRI image **(D)**.

**Figure 4:**
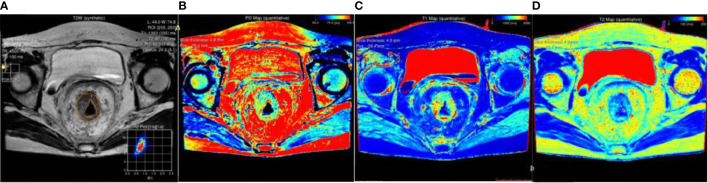
A 45-year-old female patient with rectal carcinoma. All the values were measured the on the largest slice of lesion from the SyMRI image **(A)**. PD map **(B)**, T1 map **(C)** and T2 map **(D)** images were generated.

Image analyses were performed using the MRI console with the MAGnetic resonance image Compilation (MAGiC, version 100.0.0) software. Regions of interest (ROI) were carefully and manually traced on map images on the largest cross-sectional area of the lesions. The rectal carcinoma (ROI-1) and pararectal fat space (ROI-2) were chosen for the ROI measurement respectively in each patient. In the control group, the normal intestinal wall was delineated. ROI delineation criteria: 1) tumor (ROI-1): We tried to measure the values on the largest slice of lesions (**Figures 3A, B**); 2) pararectal fat space (ROI-2): The pararectal fat region within 15mm of the tumor involving the circumferential range of the intestinal wall was delineated (**Figures 3C, D**); 3) Normal intestinal wall: Outline the normal intestinal wall in the upper or middle third of the rectum. The values for T1, T2 relaxation times and PD in each patient were measured three times, and the mean value of each ROI was used for the final analysis (**Figure 4**).

### Statistical Analysis

The data were analyzed using SPSS 25.0 software (IBM, Armonk, NY, USA) and MedCalc 11.4 (MedCalc, Mariakerke, Belgium), and *P* < 0.05 was considered statistically significant. The measurement consistency between two readers was evaluated using intraclass correlation coefficient (ICC). The ICC value was interpreted in the following way: ICC < 0.20, slight agreement; ICC = 0.21–0.40, fair agreement; ICC = 0.41–0.60, moderate agreement; ICC = 0.61–0.80, substantial agreement; ICC =0.81–1.0, almost perfect agreement. Shapiro-Wilk W-Test (n ≤ 50) and Kolmogorov-Smirnov test (n>50) were used to test the normality. One-way analysis of variance (ANOVA) was performed to test the homogeneity of variance. Measurement data following the normal distribution were expressed as mean ± standard deviation (SD), and the others were as median (first quartile, third quartile). Student t-test and Mann Whitney U test were used depending on whether the data were normal or not. Spearman’s rank correlation was performed to determine the correlation between T1, T2 and PD values of the two ROIs with the EMVI scores. The combined model was constructed using logistic regression model. The univariate receiver operating characteristic curve (ROC) analyses were performed, and areas under the curve (AUC) were compared to indicate the accurateness of the different parameters. Differences in diagnostic efficiency of single characteristic model and combined model were analyzed using the Delong test.

## Results

A total of 100 patients were ultimately included in this study. The clinical characteristics of the patients included in the study were summarized in **Table 1**. The age and gender were not statistically significant between patients with and without rectal cancer (P =0.575 and 0.795, respectively). We randomly sampled 50 patients and tested the consistency of the ROI outlined by the two radiologists. ICC values were perfect, (ICC > 0.9, 0.960-0.990)

**Table 1:** Characteristics of patients included in this study.

Characteristics	Number of patients
Mean age, years (range)	60 (24−83)
Gender	
Male	63
Female	37
pT stage	
pT1-2	21
pT3-4	79
pN stage	
pN0	48
pN1−2	52
pEMVI	
negative	42
positive	58
mrEMVI	
0	6
1	9
2	27
3	17
4	41

pEMVI, pathological extramural venous invasion; mrEMVI, magnetic resonance imaging-detected extramural venous invasion.

### The Diagnostic Performance of Different Parameters in Discriminating Rectal Cancer From Normal Rectum

**Figure 5:**
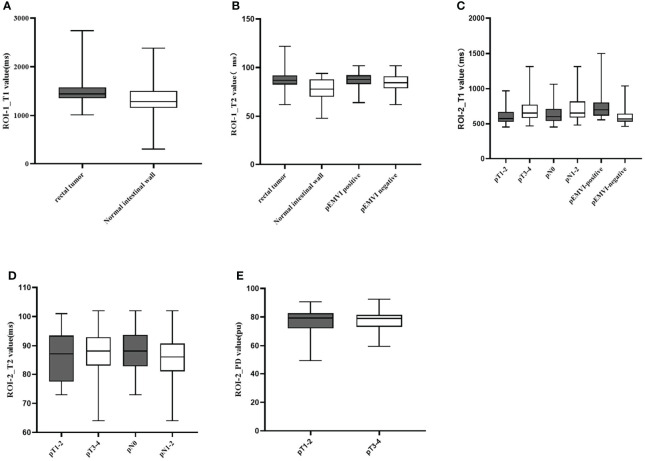
Box-and-whisker plots of T1, T2 values in ROI-1 and T1,T2,and PD values in ROI-2.T1 values of ROI-1 based on rectal tumor **(A)**; T2 values of ROI-1 based on rectal tumor and extramural venous invasion (EMVI) **(B)**; T1 values of ROI-2 based on different T stage, N stage, and EMVI **(C)**; T2 values of ROI-2 based on different T stage and N stage **(D)**; PD values of ROI-2 based on different T stage **(E)**. Significant differences were found between the two groups (all p < 0.05).

**Figure 6:**
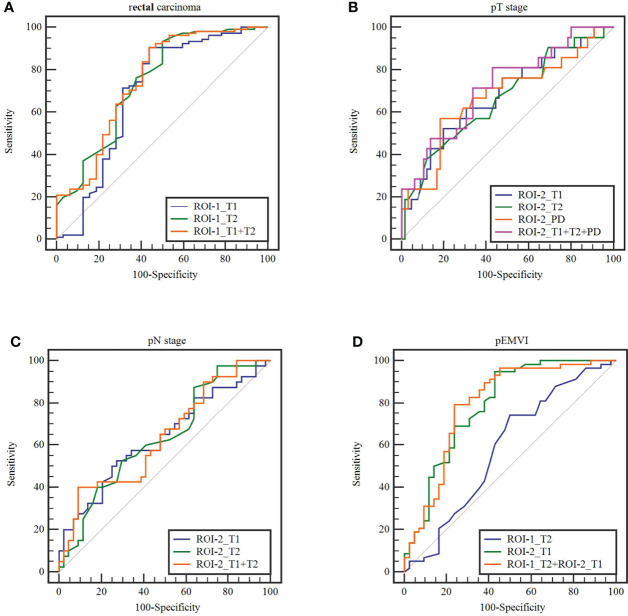
The receiver operating characteristics curves of ROI-1_T1, ROI-1_T2,ROI-2_T1,ROI-2_T2, AND ROI-2_PD values and combined model in terms of **(A)**rectal carcinoma, **(B)** T stage, **(C)** N stage and **(D)** extramural venous invasion (EMVI).

Comparison results of parameters get from syMRI between carcinoma and normal rectum were shown in **Table 2**. Compared with the normal rectal wall, the values of T1 and T2 relaxation time of the tumor were significantly higher (*P* = 0.001 and *P <*0.001, respectively). There was no statistically significant difference in the PD value (P >0.05). The AUC of T1 value and T*2* value in the tumor were AUC = 0.692, 95% CI: 0.565-0.818, *P* = 0.001 and AUC = 0.744, 95% CI: 0.639-0.850, *P*<0.001, respectively (**Figures 5A, B**, **6A**). Combining the values of TI and T2, the AUC (0.749,95% CI:0.668 to 0.819) was slightly improved.

**Table 2:** Comparison of T1, T2 and PD between rectal cancer and normal rectum.

	No.	T1 (ms)	T2 (ms)	PD (pu)
Rectal tumor	100	1442.50 (1328.25, 1556.75)	87.12 ± 8.20	79.15 (74.85, 83.45)
Normal intestinal wall	32	1288.00 (1061.50, 1514.50)	77.63 ± 10.89	74.30 (67.05, 146.80)
*P* value		0.001*	<0.001**	0.114
AUC (95% CI)		0.692 (0.565-0.818)	0.744 (0.639-0.850)	–

Data following the normal distribution are expressed as mean ± standard deviation. Otherwise, data are expressed as median (first quartile, third quartile). PD, proton density; AUC, area under the receiver operating characteristic curve; *P < 0.05; **P < 0.001.

### The Diagnostic Performance of Different Parameters for Evaluating T, N Stage and pEMVI of Rectal Cancer

The diagnostic performance of different parameters for evaluating T, N stage and pEMVI of rectal cancer are shown in **Table 3**.

**Table 3:** The indicators from syMRI for evaluating T, N stage and EMVI of rectal cancer.

Groups	ROI-1	ROI-1	ROI-1	ROI-2	ROI-2	ROI-2
	T1 (ms)	T2 (ms)	PD (pu)	T1 (ms)	T2 (ms)	PD (pu)
pT stage						
pT1-2	1455.00 (1275.50, 1634.50)	86.62 ± 8.51	79.30 (74.00, 84.60)	575.00 (505.00, 645.00)	137.00 (129.25, 144.75)	106.10 (99.65, 112.55)
pT3-4	1442.00 (1337.50, 1546.50)	87.38 ± 7.58	79.00 (74.90, 83.10)	654.00 (559.25, 748.75)	133.00 (127.25, 138.75)	99.20 (94.30, 104.10)
*P value*	0.778	0.697	0.744	0.013*	0.025*	0.017*
pN stage						
pN0	1464.50 (1321.50, 1607.50)	88.35 ± 7.70	79.25 (74.05, 84.45)	602.50 (517.75, 687.25)	136.00 (129.38, 142.63)	100.65 (95.39, 105.91)
pN1-2	1439.00 (1331.50, 1546.50)	86.00 ± 7.87	79.05 (75.03, 83.08)	654.00 (538.88, 769.13)	133.00 (125.13, 140.88)	99.30 (93.15, 105.45)
*P value*	0.576	0.171	0.754	0.035*	0.034*	0.162
pEMVI						
Negative	1428.00 (1302.25, 1553.75)	85.00 (77.50, 92.50)	76.50 (70.60, 82.40)	575.00 (506.75, 643.25)	134.55 ± 8.83	101.30 (94.10, 108.50)
Positive	1446.00 (1337.50, 1554.50)	88.00 (84.25, 91.75)	79.70 (76.23, 83.18)	685.00 (599.50, 770.50)	131.43 ± 15.52	99.50 (94.05, 104.95)
*P value*	0.297	0.002*	0.178	0.001*	0.297	0.947

Data following the normal distribution are expressed as mean ± standard deviation. Otherwise, data are expressed as median (first quartile, third quartile). ROI, region of interest;ROI-1,ROI of the rectal carcinoma;ROI-2,ROI of pararectal fat space; PD, proton density; pEMVI, pathological extramural venous invasion; *P < 0.05.

T2 value of the rectal cancer (ROI-1) were higher in the pEMVI positive group than that in the negative group (P=0.002, AUC=0.587, 95% CI:0.475- 0.692) (**Figure 5B**), but the T1 and PD values of ROI-1 had no difference between pEMVI positive and negative groups (P=0.297,0.178,respectively). The T1, T2, and PD values of ROI-1 were no difference between T stages (P=0.778, 0.697, 0.744,respectively) and N stages (P=0.576, 0.171, 0.754,respectively) of RC.

T1, T2 and PD value of the pararectal fat space (ROI-2) were effective for distinguishing both T and N stages of rectal cancer. Higher-grade stage had showed higher T1 value (P_T stage_=0.013, P_N stage_=0.035), lower T2 value (P_T stage_=0.025, P_N stage_=0.034) and lower PD value (P_T stage_=0.017). T1 value of ROI-2 were higher in the pEMVI positive group (P=0.001) (**Table 4**, **Figure 5C–E**, **6B, C**).

**Table 4:** Diagnostic performance of quantitative relaxation maps in predicting T,N stage and EMVI of rectal cancer.

Parameters	AUC (95% CI)	Sensitivity	Specificity	PPV	NPV	Cutoff (msec)
ROI-2 T1 value (pT stage)	0.681 (0.572− 0.777)	80.00%	52.38%	30.87%	90.79%	>575
ROI-2 T2 value (pT stage)	0.664 (0.554− 0.762)	87.69%	38.10%	27.35%	92.08%	≤140
ROI-2 PD value (pT stage)	0.674 (0.564− 0.771)	81.54%	57.14%	50.57%	92.09%	≤105.5
ROI-2 T1+T2+PD (pT stage)	0.718 (0.611 to 0.810)	56.92%	80.95%	44.27%	87.61%	>0.77976
ROI-2 T1 value (pN stage)	0.634 (0.522− 0.736)	72.73%	52.50%	58.56%	67.59%	>608
ROI-2 T2 value (pN stage)	0.634 (0.522− 0.737)	36.36%	87.50%	72.86%	59.83%	≤126
ROI-2 T1+T2 (pN stage)	0.638 (0.526- 0.740)	90.91%	40.00%	58.31%	82.66%	>0.42111
ROI-2 T1 value (pEMVI)	0.787 (0.693- 0.882)	94.83%	57.14%	75.34%	88.89%	>580
ROI-1T2 value (pEMVI)	0.583 (0.480− 0.681)	74.14%	50.00%	67.19%	58.34%	>84
ROI-2 T1+ROI-1_T2 (pEMVI)	0.794 (0.701− 0.882)	79.31%	76.19%	82.14%	72.73%	>0.50023

PD, proton density; pEMVI, pathological extramural venous invasion; AUC, area under the receiver operating characteristic curve; CI, confidence interval; PPV, positive predictive value; NPV, negative predictive value; ROI, region of interest; ROI-1,ROI of the rectal carcinoma; ROI-2,ROI of pararectal fat space.

The parameters with significant difference were enrolled in the combined model. pEMVI combined model showed higher AUC than the T2 value of the rectal cancer (p < 0.001). Although the combined model showed higher AUC than any single parameter in both T and N stage, but the difference of Delong test was not significant (**Figure 6**).

### Quantitative Relaxation Maps for the Ranking Correlation Between T1, T2, PD Values and mrEMVI Scores

**Figure 7:**
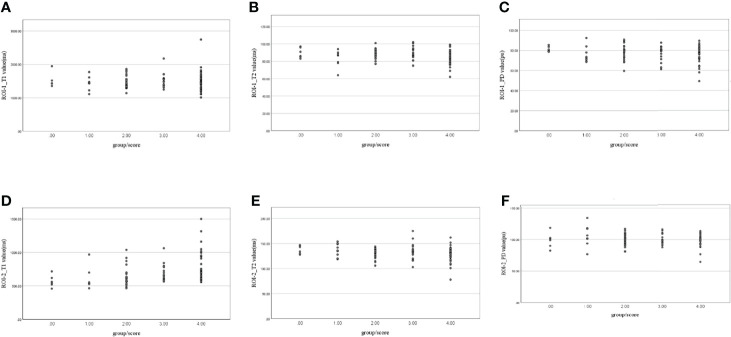
Scatter plots of the correlation between **(A)** ROI-1_T1, **(B)** ROI-1_T2, **(C)** ROI-1_PD, **(D)** ROI-2_T1, **(E)** ROI-2_T2, and **(F)** ROI-2_PD values and extramural venous invasion (EMVI) scores.

The T2 value (r=0.213, P=0.049) and PD value (r=0.354, P=0.001) of ROI-1 was correlated with EMVI score. A significant positive correlation was found between the T1 value of ROI-2 and EMVI score (r value = 0.519, P<0.001). Correlation analysis did not show any significant associations between the other parameters and EMVI scores (**Table 5** and **Figure 7**).

**Table 5:** Quantitative relaxation maps for evaluating mrEMVI of Rectal cancer.

mrEMVI scores	ROI-1	ROI-1	ROI-1	ROI-2	ROI-2	ROI-2
	T1 (ms)	T2 (ms)	PD (pu)	T1 (ms)	T2 (ms)	PD (pu)
0	551.50 (506.00- 643.25)	138.50 (129.50- 146.25)	100.35 (88.18- 106.58)	543.00 (490.00- 638.00)	143.00 (131.00- 146.50)	99.00 (86.25- 110.10)
1	541.00 (528.50- 626.00)	136.00 (124.00- 149.50)	102.10 (97.50- 117.50)	535.50 (529.25- 861.50)	136.50 (130.25- 149.25)	99.55 (89.45- 103.43)
2	601.00 (561.00- 678.00)	132.00 (125.00- 137.00)	101.60 (92.30- 107.80)	604.50 (553.00- 650.75)	133.50 (127.75- 137350)	102.70 (92.38- 107.83)
3	654.00 (605.00- 788.00)	134.00 (124.00- 141.50)	98.50 (94.40- 109.70)	650.50 (591.75- 743.25)	134.00 (123.00- 140.25)	99.60 (94.65- 110.33)
4	707.00 (625.00- 922.50)	131.00 (124.00- 137.50)	99.50 (94.10- 103.30)	691.00 (610.00- 848.00)	131.00 (126.00- 140.00)	99.50 (97.20- 104.40)
*r*	0.213	0.100	0.354	0.519	-0.148	-0.101
*P value*	0.049*	0.359	0.001*	<0.001**	0.143	0.318

PD, proton density; mrEMVI, MRI–detected extramural venous invasion; ROI, region of interest;ROI-1,ROI of the rectal carcinoma;ROI-2,ROI of pararectal fat space;*P <0.05; **P <0.001.

## Discussion

In this study, synthetic MRI was used for investigate the effect of relaxation maps on differentiating RC from healthy tissue, as well as evaluating its T, N stage and EMVI (25). It was found that T1 and T2 relaxation times of rectal adenocarcinoma were significantly higher than that of normal intestinal wall. We performed a novel analysis between the biological characteristics of rectal cancer and parameters get from pararectal fat space, and found that multi-parameters model provided a higher diagnostic efficiency to evaluate T, N stage and EMVI.

The scan time of traditional T1 mapping or T2 mapping was relatively long (21, 26, 27). Some abdominal studies (22, 23, 28, 29), for example Zhao et al. showed that SyMRI of RC did not have decreased diagnostic efficacy compared to conventional MRI, and had better diagnostic efficacy in subjective evaluation of radiologists. In this study, the SyMRI is guaranteed to have the satisfying resolution as the conventional sequence and is sufficient for diagnostic purposes (30, 31). MAGiC is a multiple-delay-multiple-echo (MDME) sequence, and quantitative values derived from the MDME sequence (15) are overall robust on 3.0 T scanners from different vendors (29). SyMRI obtains multiple relaxation weighted images through one acquisition to ensure the accurate registration in the anatomical position from different parameter images (17). T1 and T2 maps can be used to fit the quality parameters to ensure the stability of the values when drawing the ROIs (6). The values of T1, T2, and PD can effectively identify the changes of water (32), protein and collagen in tissues (6, 33).

Our study showed that the mean values of T1 and T2 relaxation time in rectal carcinoma were significantly higher than that in the normal rectal wall. Changes in T1, T2, and PD could be caused by pathological conditions (34–36). For many tissues, T1and T2 might be multi-exponential (37), T1 and T2 relaxation times are sensitive to edema, iron overload, and the presence of tissue infarcts and scarring (38). The tumor tissue might lead to increased blood flow and vascular permeability (39). When the rectal wall was infiltrated by cancer cells, the number of cells increased with a loose intercellular junction and the water content of interstitial space increased. Then, the longitudinal relaxation time and transverse relaxation time were prolonged, the values of T1 and T2 relaxation time increased.

The main role of MRI in rectal cancer is not to qualitative diagnosis, but to quantitative diagnosis, such as T, N stage and EMVI. Firstly, we used parameters of tumor (ROI-1) for quantitative diagnosis, only T2 values were statistically significant for identifying the presence of pEMVI, and T2 values were increased in the EMVI-positive group. The previous study (40) found that T1 and T2 values of tumor were all useful for predicting prognostic factors of RC. They think highly aggressive RC have lower T1 value and T2 value, which is different from our results, it maybe because of different ROI drawing method. Meanwhile, Some studies have suggested that poorly differentiated carcinoma (40) have lower T2 values, and mucinous adenocarcinoma(MA) (41) have higher T2 values. In this study, mucinous adenocarcinoma was excluded, and most of the tumors were moderately to highly differentiated rectal cancer (only 6 cases were poorly differentiated), and the tumors were mostly glandular components, which may lead to no differences of parameters in the T and N stage. In contrast, EMVI appeared different maybe because it is an independent predictor of prognosis. Furthermore, EMVI should be more researched.

In this study, the parameters of pararectal fat space were used to evaluate prognostic factors of RC, which can directly show tumor invasiveness. The results showed the higher-grade pathological stage of tumor had higher T1 value, lower T2 value and lower PD value. This may be due to the replacement of fat by tumor tissue, which prolongs the longitudinal relaxation time (T1) and shortens the transverse relaxation time (T2), consistent with the performance observed on the MR images. The quantitative T2 relaxation time is considered as a reliable and reproducible quantitative biomarker that can reflect the flow water content in different tissues (22). And quantification of the T1 value has been an essential approach of myocardial diffuse fibrosis assessment (19, 20, 42, 43) But it should be noted their change should be influenced by tissue characteristic. Meanwhile, the combined model had better diagnostic efficacy, higher specificity (80.95%) for T-staging and higher sensitivity (90.91%) for N-staging, it suggested that a combined model is preferred when applying multi-quantitative parameters for grading RC. So, the quantitative relaxation time of pararectal fat space could provide benefits in clinical work about T, N stage of RC without extended scanning times and complex post-processes, as well as it could be obtained in a single acquisition.

(44)Another attractive finding was a significant positive correlation between the T1 value of pararectal fat space, the T1, PD value of tumor and the EMVI score. EMVI has been considered as an independent prognostic factor for rectal carcinoma. Patients with proven vascular invasion have shorter progression-free survival and overall survival (5, 45–47). pEMVI can only be obtained postoperatively and cannot be scored (48). Currently, EMVI can be detected preoperatively by MRI used for rectal cancer stage. But the mrEMVI score may be influenced by the subjective diagnostic experience and scanning parameters. Objective quantitative evaluation is more conducive to improve diagnostic accuracy (49). Gursoy et al. (50) had suggested that the ADC value was associated with EMVI diagnosis of rectal adenocarcinoma. We conducted further inter-group comparison of patients in 5 groups with different EMVI scores. With the increasing EMVI score, the value of T1 relaxation time of pararectal fat space also increased, and the T1 value of pararectal fat space had more diagnostic performance. T1 relaxation is the energy transfer from internal proton to external proton, while T2 relaxation is the energy transfer within the protons, and T1 relaxation time is longer than T2 relaxation time in all tissues for the same target region (44). Meanwhile, the T1 relaxation time of fat is shorter than tumor tissue, and the T2 relaxation time of fat is longer than tumor tissue. As the tumor cells invade the pararectal fat space, it means the replacement of pararectal fat by tumor tissue, which prolongs the T1 relaxation time and shortens the T2 relaxation time, this is consistent with the performance on the MR images, and this change becomes more significant with the progress of tumor, especially on the T1 weighted images. Therefore, it is speculated that the T1 relaxation time might be more sensitive to detect the EMVI. Quantitative mapping without administration of a paramagnetic contrast agent may become an essential tool to understand rectal tissue pathology and its prognostic implications. Besides, quantifying tissue characteristics, T1 mapping makes it feasible to follow longitudinal time changes, which are necessary as a novel biomarker in clinic. Thus, the synthetic MRI could be a quantitative method to assist conventional MRI and increase the reference parameters for diagnosis and prognosis, just like the tumor markers obtained from blood.

There are some limitations in this study. Firstly, this is a single-center study, and multi-center and multi-regional studies should be carried out in the future. Though the same MR device was used for scanning in our research to avoid differences in variable factors to some extent, there were limitations for subsequent reproducible research and promotion. A larger sample is also needed to determine the range of T1 and T2 values for rectal carcinoma and normal tissue. Secondly, patients with mucinous adenocarcinoma were excluded from this study, which is a distinct subtype (51) and is characterized by abundant mucinous components that comprise of at least 50% of the tumor volume. Thirdly, in order to ensure that both ROIs of pararectal fat space and tumor could be obtained in the same cohort, only the patients with mid-upper rectal cancer were selected, because the surrounding fat is always absent in the lower rectal cancer. Further studies should be performed including the lower rectal cancer. Fourthly, there were relatively few rectal carcinoma with an EMVI score 0. Patients with rectal carcinoma are usually found in the advanced stage, so the morphology of lesions is irregular, and the lesions are mostly annular wall growth with more peripheral blood vessels invaded, as a result the mrEMVI were usually scored ≥ 2 when the tumor be discovered. So, maybe there was selection bias in the experimental group. Fifthly, only 6 patients with poorly differentiated adenocarcinoma were enrolled in this study, so the study about tumor differentiation was not performed.

## Conclusion

Synthetic MRI can provide multi-parameters quantitative maps with a easier measurement and shorter acquisition time compared with conventional MRI. The measurement of multi-parametric quantitative values contributes to diagnose the rectal cancer and evaluate T, N stage and EMVI. It has the potential to be used as a preoperative diagnostic and grading technique of rectal carcinoma.

## Data Availability Statement

The data supporting the conclusions of this study are available from the corresponding author YL on request. Requests to access the datasets should be directed to YL, liuyicmu@sina.cn.

## Ethics Statement

The studies involving human participants were reviewed and approved by The First Hospital of China Medical University. Written informed consent for participation was not required for this study in accordance with the national legislation and the institutional requirements. Written informed consent was obtained from the individual(s) for the publication of any potentially identifiable images or data included in this article.

## Author Contributions

KZ carried out the main experimental operation and wrote the paper. ZC offered statistical analysis help and revised the manuscript together with KZ. LC is responsible for article writing together. JZ collected the patients together. YL and JC jointly designed the experimental scheme. All authors contributed to the article and approved the submitted version.

## Conflict of Interest

The authors declare that the research was conducted in the absence of any commercial or financial relationships that could be construed as a potential conflict of interest.

## Publisher’s Note

All claims expressed in this article are solely those of the authors and do not necessarily represent those of their affiliated organizations, or those of the publisher, the editors and the reviewers. Any product that may be evaluated in this article, or claim that may be made by its manufacturer, is not guaranteed or endorsed by the publisher.
